# Small Satellite Tools for High-Resolution Infrared Fire Monitoring

**DOI:** 10.3390/jimaging8020049

**Published:** 2022-02-16

**Authors:** Christian Fischer, Winfried Halle, Thomas Säuberlich, Olaf Frauenberger, Maik Hartmann, Dieter Oertel, Thomas Terzibaschian

**Affiliations:** 1 German Aerospace Center (DLR), Institute of Optical Sensor Systems, Rutherfordstr. 2, 12489 Berlin, Germany; winfried.halle@dlr.de (W.H.); saeuberl@dlr.de (T.S.); thomas.terzibaschian@dlr.de (T.T.); 2 DLR, Institute for Solar-Terrestrial Physics, Woldegker Chaussee 35, 17235 Neustrelitz, Germany; olaf.frauenberger@dlr.de; 3 Astro- und Feinwerktechnik Adlershof GmbH, Albert-Einstein-Str. 12, 12489 Berlin, Germany; m.hartmann@astrofein.com (M.H.); d.oertel@astrofein.com (D.O.)

**Keywords:** high temperature event, fire detection, infrared sensor system, small satellite, FireBIRD

## Abstract

Space-borne infrared remote sensing specifically for the detection and characterization of fires has a long history in the DLR Institute of Optical Sensor Systems. In the year 2001, the first DLR experimental satellite, Bi-spectral Infrared Detection (BIRD), was launched after an intensive test period with cooled IR sensor systems on airborne systems. The main basis for the development of the FireBIRD mission with the two satellites, Technology Erprobungsträger No 1 (TET-1) and Bi-spectral-Infrared Optical System (BIROS), was the already space-proven sensor and satellite technology with successfully tested algorithms for fire detection and quantification in the form of the so-called fire radiation power (FRP). This paper summarizes the development principles for the IR sensor system of FireBIRD and the most critical design elements of the TET-1 and BIROS satellites, especially concerning the attitude control system—all very essential tools for high-resolution infrared fire monitoring. Key innovative tools necessary to increase the agility of small IR satellites are discussed.

## 1. Introduction

The DLR initiative in building small satellites was initiated in 1994 by the Phase A study “Fire Recognition System for Small Satellites” (FIRES). Based on this study, the DLR started the initiative of “cost-effective Earth Observation Missions”. The rapid progress of the ongoing technology developments that led to further miniaturization of engineering components and to new micro-optoelectronic technologies for sensors and instruments allowed for the design of a small satellite. The Bi-spectral Infrared Detection (BIRD) satellite, launched in 2001, was the first small satellite to be designed, developed, tested, and successfully operated in orbit by the German Aerospace Center (Deutsches Zentrum für Luft- und Raumfahrt e.V. DLR) [1]. BIRD demonstrated not only the validity of a design to cost approach, widely using components-off-the-shelf (COTS) parts, but also the in-orbit application of cooled infrared sensors for the detection of high-temperature events (HTE). The mission has also shown the feasibility and usefulness of prospective operational small satellite systems. This included on-board orbit determination as well as on-board data processing with thematic data reduction and generation of thematic maps. Thus, the BIRD mission demonstrated the substantial potential for detection and quantitative assessment of high-temperature events by bi-spectral infrared push-broom sensors [2]. Based on requirements defined within the concept of “affordable space missions”—a study initiated by the DLR program directorate “Earth Observation” in 2005—the BIRD mission focused on specific Earth Observation phenomena.

In the following years, TET-1 and BIROS were developed by evolving the modular satellite bus approach of BIRD. The satellite launch of TET-1 took place from Baikonur in 2012. After the first phase of technology testing in 2012, TET-1 was successfully operated for FireBIRD from October 2013 until mid-2019. BIROS was successfully launched in June 2016 from the Indian space center Sriharikota Range and was in continuous operation until the termination of the FireBIRD mission at the end of 2020. For over five and seven years, respectively, both sensor systems delivered high-resolution infrared image data for quantitative assessment of high-temperature events, including wildfire detection and monitoring.

The intent of this paper is to show how the main payloads and essential components of the TET-1 and BIROS small satellite buses were developed based on the requirements of the FireBIRD mission.

## 2. FireBIRD Primary Mission Requirements

The primary objectives of the FireBIRD mission are infrared remote sensing of high-temperature events (HTE). These objectives are briefly described in the FireBIRD Mission Requirement Document [3] by the following items:

- Establishment of a pre-operational fire monitoring constellation consisting of TET-1 and BIROS;
- Installation and testing of different operational modes for continuous monitoring of selected regions;
- Testing of different overflight formations and maneuvers to demonstrate the agility of the satellites;
- Survey of vegetation fires, peat fires, and coal seam fires and derivation of important climate-related variables of these fires, in particular the three parameters of the Essential Climate Variable (ECV) “Fire Disturbance”:
  o precise geographic localization of “Active Fires” fires
  o estimation of the upwelling radiant power of the fire, i.e., estimation of the “Fire Radiative Power” (FRP); and
  o fire-area mapping parameters, e.g., the “Burned Area”—for daytime data takes, as well as deriving other fire attributes, such as the strength and length of forest and bush fire fronts (“fire line strength and length”), and further:
- Observation and assessment of volcanic and geo-thermal activities, especially for:
  o monitoring of selected active volcanoes;
  o detection of thermal precursors of eruptions;
  o monitoring and thermal mapping of volcanic eruptions; and
  o mapping and detection of thermal changes in geo-thermal active areas.
- Validation, i.e., critical evaluation of accuracy:
  o of fire attributes, such as the FRP, effective fire area, effective fire temperature, and others in the spatial mapping of the detected HTE; and
  o by cross validation of fire emission data, especially those from spatially coarser resolution satellite sensors, which detect large fires at shorter time intervals than FireBIRD.

The recommended Noise Equivalent Difference Temperature (NEΔT) of the mid-infrared (MIR) band and the thermal infrared (TIR) band of a space-borne sensor for high-resolution IR fire monitoring is 0.3–0.5 K and 0.2 K, respectively, for a 300 K blackbody source [4]. The BIRD mission demonstrated that these NEDT numbers can be achieved by IR sensors equipped with cooled photo detectors in the MIR and TIR bands. The NEΔT represents the radiometric measurement accuracy.

## 3. The FireBIRD Main Payload

The design concept of the FireBIRD main payload had to fulfill the FireBIRD mission requirements and it refers to the proven BIRD sensor system, which consists of [4]:

- a two-channel infrared Hot Spot Recognition Sensor system (HSRS); and
- a Wide-Angle Optoelectronic Stereo Scanner adapted to the BIRD mission (WAOSS-B).

The HSRS is a push-broom scanner with spectral bands in the mid-infrared (MIR) and thermal infrared (TIR) spectral ranges. The detectors of the bi-spectral sensor are two Cadmium Mercury Telluride (CdHgTe) photodiode line arrays—with an identical layout in the MIR and the TIR—comprising 2 × 512 elements each in a staggered structure [5]. The WAOSS was initially developed for the Mars-96 mission and its Flight Spare Model was modified to be used as BIRD’s visible–near IR (VIS-NIR) push-broom camera. Table 1 shows the main characteristics of the BIRD sensor system.

The HSRS sensor head components of both of its spectral channels are based on identical technologies to provide good pixel co-alignment. Both spectral channels have the same optical layout but with different wavelength-adapted lens coatings. Radiometric investigations of thermal anomalies require (a) a large dynamic range to not be saturated by high-temperature events (HTE) occupying a large part of a pixel and (b) a high signal-to-noise ratio to be able to observe small thermal anomalies and detect small sub-pixel HTE. A unique feature of the BIRD MIR and TIR sensor channels is that the respective integration times can be adjusted to the maximum radiance temperature of the scene. This capability, controlled in real time, is related to one of the most important sensor features, namely maintaining an adequate signal-to-noise ratio over ambient pixels while providing unsaturated data over pixels exposed to even the most intense fire events. If on-board processing of the HSRS data indicates that detector elements are saturated during the first exposure (or are close to saturation such that they may be outside the range for which the channel calibration is most accurate), then a second exposure is performed with a reduced integration time [6,7].

The FireBIRD main payloads, i.e., the multi-spectral camera systems of TET-1 and BIROS, are in their bi-spectral IR sensors nearly identical with BIRD’s Hot Spot Recognition System, but they comprise a new VIS-NIR CCD push-broom camera whose entrance lens has a focal length of 90.6 mm, which is quite different to the 21.65 mm focal length WAOSS used in the BIRD mission (see Table 1). This allows for a ground sampling distance (GSD) of up to 40 m in the visible and near-IR bands, giving us the opportunity to support the sub-pixel technique of the bi-spectral method [8]. The bi-spectral cameras of the FireBIRD satellites TET-1 and BIROS were developed and built at the DLR Institute for Optical Sensor Systems in Berlin-Adlershof. 

**Figure 1:**
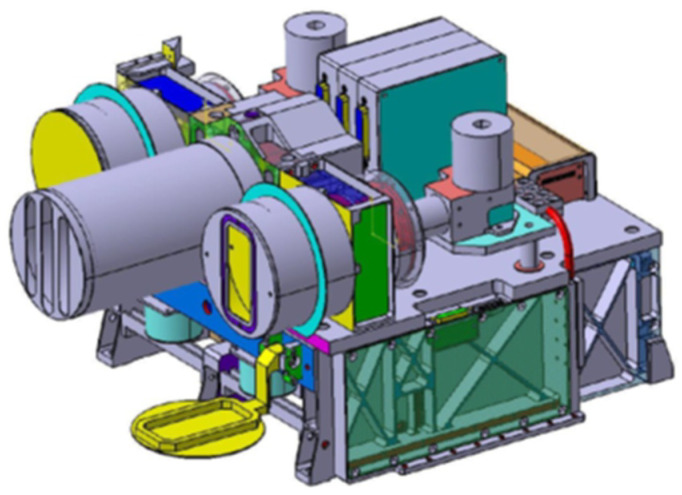
Perspective view of the sensor system of BIROS with the VIS-NIR camera in the center and the MIR and TIR sensors arranged to the right and left of it, respectively.

The bi-spectral IR sensor and the VIS-NIR camera of the BIROS multispectral camera system are located on a common mechanical carrier console as shown in Figure 1. In the center of the camera system is the lens of the VIS-NIR camera in whose focal plane three Charge-Coupled Device (CCD) lines with their respective spectral filters are arranged. Behind this lens, three so-called Front End Electronic (FEE) units for the two visible spectral bands and the near-IR band of the VIS-NIR camera are clearly visible as a compact block. To the right of the VIS-NIR lens is the lens and the so-called Integrated Detector Cooler Assembly (IDCA) of the MIR band of the bi-spectral IR sensor. To the left of the VIS-NIR lens is the lens and IDCA of the TIR band of the bi-spectral IR sensor of this camera system. The two FEE units of the bi-spectral sensor are mounted on the side of the carrier console and connected to the respective IDCA via a cable connection (shown in red for the MIR camera in Figure 1).

The three CCD line arrays of the TET-1 and BIROS VIS-NIR cameras have a spacing of 10.1 mm to each other, which—in combination with the focal length of 90.6 mm—results in an angle of 6.4 degrees between the forward- and nadir-looking lines and the nadir- and backward-looking lines.

Since there are significant differences in the operation of the bi-spectral sensor compared with the VIS-NIR camera, it makes sense to describe them separately.

Table 2 contains features and parameters of the multi-spectral camera system of TET-1 and BIROS.

**Figure 2:**
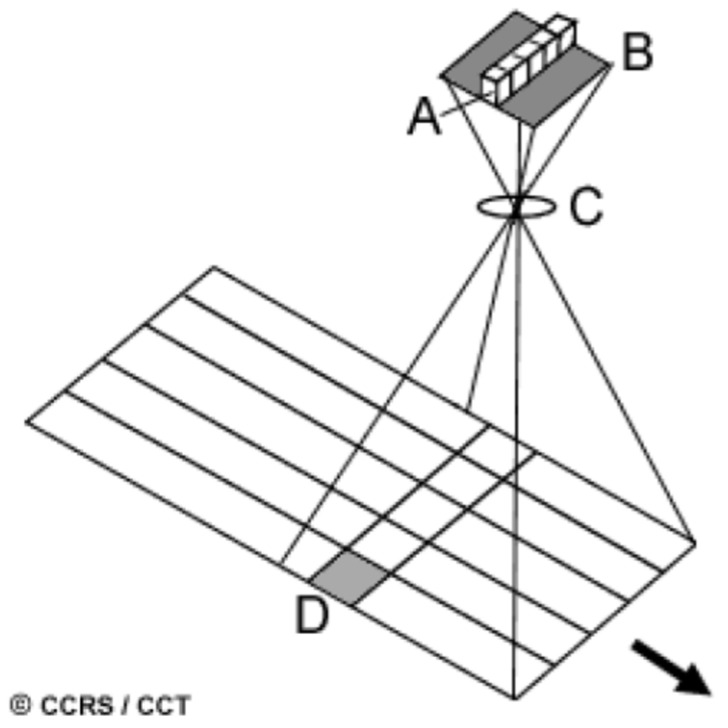
Working principle of a push-broom camera. The detector line A is located on the focal plane B of the camera lens C. The arrangement A–B–C moves on a carrier (aircraft, satellite) in the direction of the arrow over the ground, where a ground pixel D is represented. The pixel dwell time corresponds to the time interval in which a distance is flown over and is equal to the edge length of a pixel (in the direction of movement).

The multi-spectral camera systems of the FireBIRD satellites, i.e., the main payloads of the TET-1 and the BIROS satellites, shown in Figure 2, work according to the push-broom principle: The longitudinal axes of the lines are perpendicular to the forward motion of the satellite, as shown in Figure 2; and

- The complete image scanning of the Earth takes place in one acquisition swath if the detector lines are read out with the pixel dwell time Tdw = GSD/V0, where:
o
the Ground Sampling Distance (GSD) is the sampling step width on the ground; and
o
V0 is the projection of the velocity vector of the satellite on the Earth’s surface, i.e., the velocity with which the satellite foot point moves over the Earth.

### 3.1. Bi-spectral Sensors of TET-1 and BIROS

Mechanical flaps are mounted in front of the MIR and TIR lenses of the bi-spectral sensor, which close the lens for the radiometric on-board calibration of these cameras as shown on the left for the TIR camera in Figure 1. During Earth imaging, the flap is open as shown on the right in Figure 1 for the MIR camera.

Comments:

(1) The symbolic illustration of the flap positions in front of the MIR and TIR lenses chosen for Figure 1, however, does not correspond to the principal mode of their operation, which is characterized by both flaps being open or closed at the same time.
(2) Tables and images from various original sources are used in this section, in some of which different abbreviations are used for the thermal and mid-IR wavelength ranges, such as:
  - LW or LWIR (Long-Wave IR) for thermal IR (TIR); and
  - MW or MWIR (Mid-Wave IR) for mid-IR (MIR).

The detectors of the bi-spectral IR sensors of BIRD, TET-1, and BIROS are cooled Cadmium Mercury Telluride (CdHgTe) detector line arrays whose lines have an identical geometrical structure.

In these detectors, two 512-element-long lines are arranged close to parallel to each other on a CdHgTe semiconductor chip in such a way that the centers of the detector elements are shifted by exactly half the distance between the elements (in the line direction). These so-called staggered line arrays allow for an improvement of the geometrical resolution. The function of staggered line arrays is well explained and illustrated in [7] on the basis of airborne IR data, and it is described in physical–mathematical terms in [9].

A data sheet for the MIR and TIR detectors used in BIRD, TET-1, and BIROS is shown in Table 3 as a copy of a document (BIRD IR DETECTOR PROPOSAL) supplied in 1996 by the British company GEC Marconi for the BIRD project.

**Figure 3:**
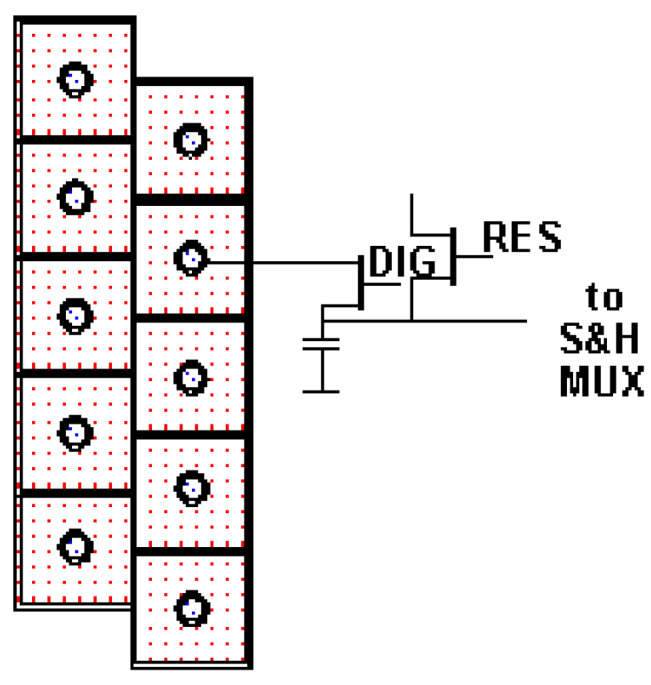
Staggered structure of the BIRD MIR and TIR detectors, which are also used in the IR sensors of TET-1 and BIROS (only 2 × 5 elements of a total of 2 × 512 elements along with the read-out circuit of one detector element are shown here) [6].

Figure 3 shows 2 × 5 elements of the staggered structure of the CdHgTe line array detectors used in the BIRD Hot Spot Recognition System (HSRS), which are also used in the IR sensors of TET-1 and BIROS [6].

#### 3.1.1. Design and Cooling of the Bi-Spectral Sensor Heads

**Figure 4:**
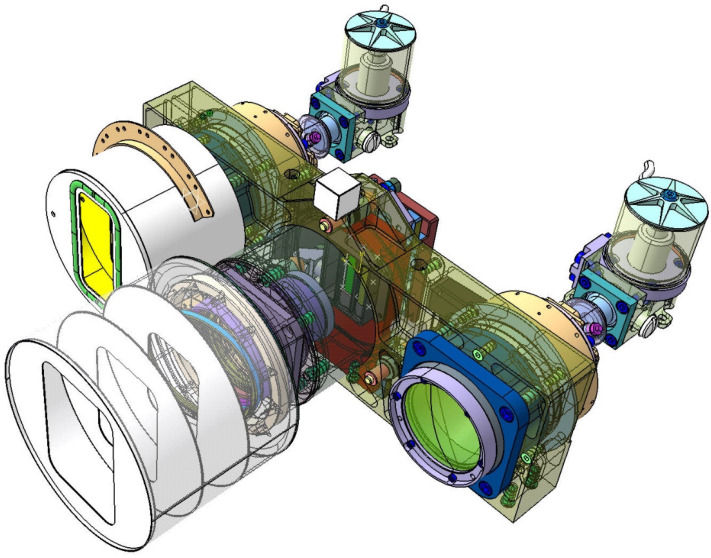
Semi-transparent view of the three sensor heads of the BIROS main payload, comprising: the MIR sensor head with its compact entrance lens and its Integrated Detector Cooler Assembly (IDCA) on the right, the TIR sensor head with its entrance lens (plus baffle in its housing) and its IDCA on the left, and the VIS-NIR sensor head with its entrance lens (plus open baffle) and three spectral filters in front of the VIS-NIR focal plane, which is equipped with three CCD linear arrays, in the center.

Figure 4 shows a semi-transparent perspective view of the three sensor heads of the BIROS main payload, i.e., the multi-spectral camera system.

On the right side of Figure 4 is shown the MIR sensor head with its compact entrance lens and its detector cooler assembly, the so-called Integrated Detector Cooler Assembly (IDCA). On the left side of Figure 4 is shown the TIR sensor head with the baffle attached to its entrance lens and its IDCA. In this view, both IR sensor heads are missing the flaps for radiometric on-board calibration, which are shown in Figure 1.

In the center of Figure 4 can be seen the VIS-NIR sensor head, which is described in the following subsection.

**Figure 5:**
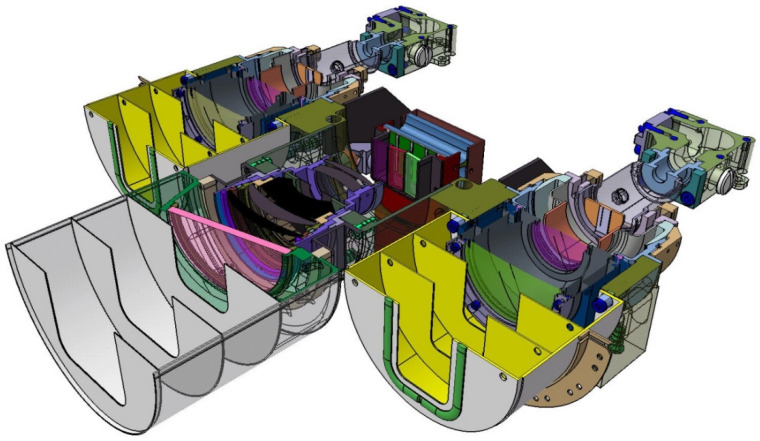
Sectional view of the sensor heads of the BIROS main payload, providing a detailed insight into its opto-mechanical interior with: the MIR sensor head with its entrance lens (plus baffle) and its IDCA on the right, the TIR sensor head with its entrance lens (plus baffle) and its IDCA on the left, and the VIS-NIR sensor head with its entrance lens (plus baffle) and three spectral filters in front of the VIS-NIR focal plane, which is equipped with three CCD linear arrays, in the center.

Figure 5 shows a sectional view of the sensor heads of the BIROS main payload, providing a detailed insight into its opto-mechanical interior. Among other things, one can see:

- on the right and left the yellow-colored so-called baffles, which protect the entrance optics of the MIR and TIR sensor head from stray light, and
- the gray-colored Dewar vessels with their brown and gray input windows, behind which the chips of the CMT line detectors, represented by two small circles, are “flying” in the focal plane of the input lenses.

Comments on Figure 5:

(1) The use of the term “flying” is due to the fact that the cold fingers of the small refrigeration machines are not shown in this sectional view, but the CMT detector chips are mounted on the tips of the two cold fingers.
(2) Numerous details of the VIS-NIR camera—shown in the center of Figure 5—will be discussed further below.

**Figure 6:**
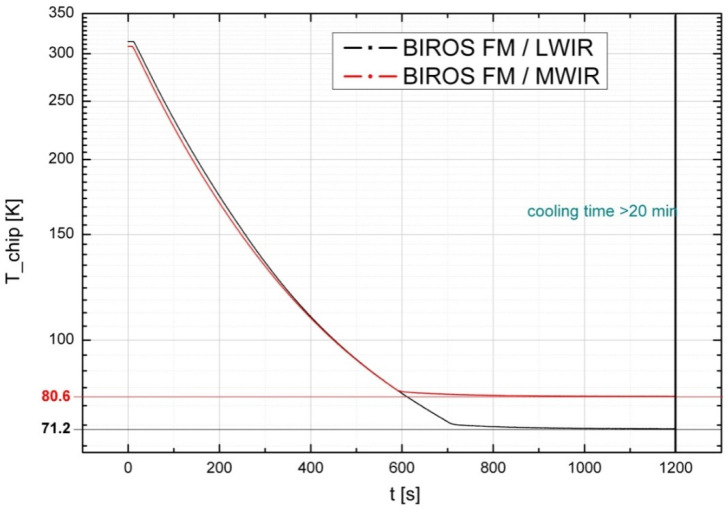
Time diagrams of the cooling processes of the BIROS MIR/MWIR and TIR/LWIR sensor heads.

The MIR detector is cooled to 80.6 K (−192.4 °C) by means of its small refrigeration machine, and the TIR detector is cooled to 71.2 K (−201.8 °C) by its small refrigeration machine. Figure 6 shows the time diagram of the cooling processes of the BIROS MIR and TIR sensor heads.

To ensure stable in-flight working temperatures of the CMT detectors from the beginning of each data acquisition cycle, the small Stirling coolers are switched on about 20 min before the start of each Earth observation data registration.

#### 3.1.2. Spatio-Temporal Sampling of the FireBIRD Bi-Spectral IR Sensors

The MIR and TIR line arrays of the two sensor heads are read out at a rate corresponding to the pixel dwell time Tdw = 25.4 ms, as shown in Table 2. Thus, a scanning step width of 178 m is achieved in the direction of flight, although the edge length of a pixel on the ground is twice as large, at 356 m. By offsetting the two “staggered linear arrays” (see Figure 3, term: “element stagger, line to line”) arranged on the focal plane of both the MIR and the TIR spectral bands by 15 µm, a scanning step size of ~178 m is also achieved perpendicular to the direction of flight, although the edge length of a pixel on the ground of 356 m is also twice as large in this direction.

A special feature of BIROS’s bi-spectral sensors is that the exposure times (or integration times) of their CdHgTe detectors can be:

- varied via ground station command; and
- adjusted in real time to the signal strength.

The latter means that during the used sampling time of 25.4 ms, the following processes take place automatically in the electronics close to the detector in the MIR and TIR sensor heads:

(a) If all 2 × 512 signal levels of the respective staggered line array are sufficiently far away from the saturation level at the commanded integration time (T int-com), which occurs in the first quarter of the sampling period of 25.4 ms, then no second exposure takes place within this sampling period.
(b) If, however, even one of the 2 × 512 detecter element signal levels of the respective staggered line array is within the range of the saturation level at the commanded integration time Tint-com, which is triggered in the first quarter of the scanning period of 25.4 ms, a further exposure is carried out in the second half of the sampling period with an integration time shortened by a factor of 30 compared with T int-com. For the elements of the detector lines where the signal level was in the saturation range during the exposure with integration time T int-com, the digitized pixel samples are appended (at the end) to the data from the first sampling with T int-com.

The data frame of the read-out digitized data of the detector lines is structured in such a way that it can accommodate the larger number of samples occurring in case (b) with exact assignment to the respective detector element.

This “intelligent” procedure used in the bi-spectral IR sensor of BIROS for the significant extension of the radiometric dynamic range by means of two successive exposures with different integration times during one sampling cycle has already been (i) successfully tested in the Advanced BIRD Airborne Simulator (ABAS) [6], (ii) used in the Hot Spot Recognition System (HSRS) of BIRD, and (iii) used in the bi-spectral sensor of TET-1. It is a unique selling point in IR sensor development for the detection and evaluation of high-temperature events. This feature permits us to achieve saturation temperatures of pixels in the MIR band of 650 K (+ 377 °C) and in the TIR band of ~ 600 K (~+327 °C), and it allows for a 20% occupation of the IR pixels of BIROS (with an area of 356 m × 356 m = 126,736 m^2^ = 12.7 Ha) by a HTE with a temperature of 1000 K (+727 °C). In other words, a very extensive fire with a temperature of 1000 K filling 0.2 × 12.7 Ha = 2.54 Ha in a BIROS IR pixel can be registered without saturation and subsequently energetically evaluated.

#### 3.1.3. Radiometric On-Board Calibration of the FireBIRD Bi-Spectral IR Sensors

The detection of high-temperature events (HTE) from space requires the use of infrared sensors, which measure the radiated power of HTEs in a reproducible and highly stable way. From this bi-spectrally measured radiated power, for example, the so-called effective temperatures can be derived [8]. In order to guarantee the stability of the measured values (i) under different “environmental influences” on the sensor system in space (e.g., different angles of solar incidence), and (ii) considering the properties of the detector system used, the measurement signals obtained from the bi-spectral sensors must be radiometrically cleaned from interfering signal components. For this purpose, the FireBIRD IR sensors must be radiometrically calibrated on board at certain time intervals. This is conducted by measuring reference radiation sources that are periodically swiveled into the field of view of the IR sensors. In the wavelength range of >3.5 µm, blackbody radiators are used for this purpose.

A special feature of IR small satellite missions is that the IR sensors with their cooling systems cannot be in operation continuously for power consumption reasons. This means that data acquisitions can be taken over selected target areas only, for which the detectors of the IR sensors have to be repeatedly cooled down and temperature-stabilized, as described above.

In the CdHgTe detectors used in BIRD, TET-1, and BIROS, which were developed in the 1990s, both the signal offset and the sensitivity of the detector elements vary after each cooling process. These variations must be measured on-board radiometrically to avoid spurious artifacts in the processed image data products. Therefore, it is essential for IR sensors operated in space with cooled detectors, especially due to their high radiometric sensitivity, to apply a radiometric on-board calibration procedure, which radiometrically characterizes the sensor system after each cooling process of the detectors and at the same time also considers the current thermal state of the system.

With the help of the flap arrangement used in front of the IR sensor heads for radiometric on-board calibration, as illustrated in Figure 1, both detector-specific changes and thermally different states of the overall system (optics, housing, etc.) can be considered. Each of these flaps is equipped with a heated blackbody radiator on its side that closes the objective. The temperature of these blackbodies can be varied via a surface-heating foil mounted on their rear sides. After the detectors have cooled down (see Figure 6), which takes place before each data take in a target area, the flaps are opened by means of stepper motors. The flaps close the optical inputs of the IR sensor heads after the end of each scene data acquisition (also to protect them from contamination), and the radiometric on-board calibration procedure is then performed, since the flaps (and the blackbodies mounted on them) are cooled down to a minimum temperature in the open state, thus enabling the radiometric correctable range of the sensor to have low temperatures.

With the aid of the surface-heating foils on the rear sides of the blackbodies, the following procedures are conducted:

- ramp-like increasing temperature curves are used for the blackbodies;
- continuous measurements of the blackbody radiation by the IR sensors; and
- temperature values of the temperature sensors integrated in the flap are recorded for each sampled measurement step of the IR sensor.

These measured temperatures of both flap blackbodies are input variables for the radiometric calibration calculation.

The output variables of this calibration procedure are the offset and sensitivity coefficients for each detector element of the MIR and TIR sensors. These coefficients, calculated for each detector element, are used to computationally convert the scene measurement data obtained as digitized raw signal levels into radiance values. Among other things, the current cooling state of the detector and the thermal state of the sensor system are further input variables for these detector-element-related calibration coefficients.

The application of these continuously updated and detector-element-related calibration coefficients to the digitized raw signal levels of the recorded scene data thus also has the effect of cleaning the derived radiance values from possible temporal variations in the thermal self-radiation components of the IR sensor heads.

The application of the detector-element-related offset and sensitivity coefficients to the scene measurement data is accomplished by the BIROS radiometric processor.

#### 3.1.4. Radiometric Processor of the BIROS Bi-Spectral IR Sensor

For availability and cost reasons, for TET-1 and BIROS, IR detector line arrays identical to those in BIRD were used. The sensitivities of the individual detector elements of these so-called “staggered linear arrays” of type E3437 (MIR) and E3438 (TIR) vary strongly because they correspond to the technological state of the art of the CdHgTe detector technology of the second half of the 1990s.

Therefore, in order to obtain scientifically usable and high-resolution radiometric image data from the bi-spectral IR sensors, the raw scene data must be converted into radiance values using the detector-element-related offset and sensitivity coefficients, i.e., for each detector element of the staggered linear arrays used in the IR sensor, obtained from the radiometric calibration measurements:

(a) in the laboratory from external blackbody measurements; and
(b) on board with blackbody measurements using the closed flaps.

As before for TET-1, for BIROS an improved radiometric processor was implemented, which essentially performs the following tasks:

- Reading in and checking the consistency and completeness of the calibration data, the scene raw image data, and the “House Keeping (HK)” data;
- Determining the detector-element-related offset and sensitivity coefficients from the on-board calibration measurements for the MIR and TIR CdHgTe lines;
- Calculating radiance values from the in-flight scene measurements for each detector element, including those elements where the signal level at the exposure with integration time Tint-com is in the saturation range, and, therefore, a second exposure with a shortened integration time is automatically performed within the same sampling period, and then—by “on-board data handling (OBDH)” —appending these digitized pixel samples of the so-called hot pixels to the data from the first exposure (at the end);
- Determining insensitive detector elements, so-called “dead pixels”; and
- Detecting and correcting (residual) striping artifacts.

The procedures described here, which run automatically in the radiometric processor of the BIROS bi-spectral sensors, were tested and further developed in the commissioning phase of BIROS (2016/2017) on numerous scenes with different mean temperatures. This applies in particular to the procedures for reducing (residual) striping structures, which were iteratively and successively improved by optimizing the operating points of the detector lines with the help of so-called “software uploads” during the commissioning phase of BIROS.

**Figure 7:**
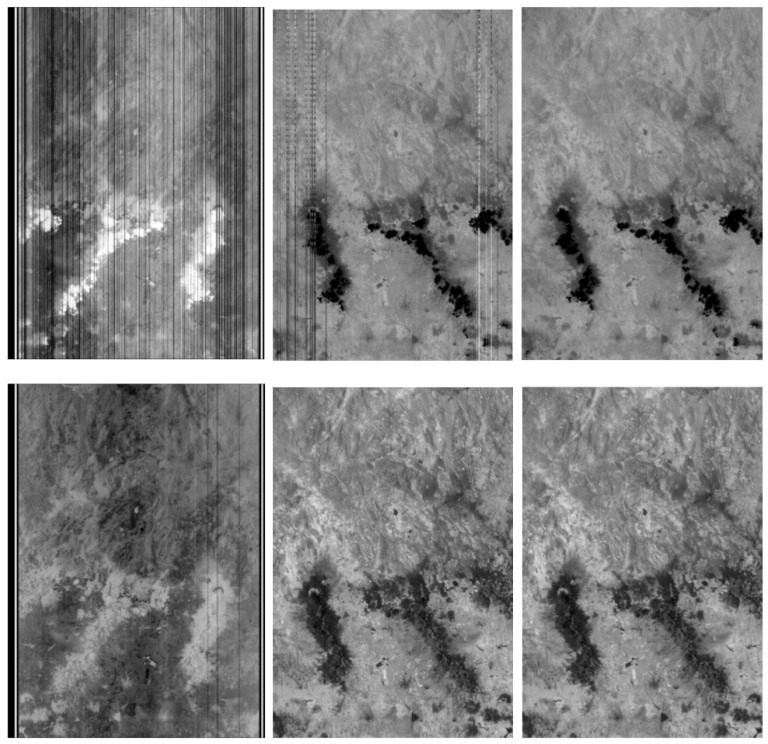
BIROS IR images of a normal-temperature scene around Mexico City on 5 May 2017: the image fragments from the BIROS TIR band in the top row and the image fragments from the BIROS MIR band in the bottom row; in both cases, the raw image fragments are shown on the left, the image fragments after the linear radiometric correction are shown in the middle, and the image fragments after the (residual) strip correction are shown on the right.

Figure 7 shows BIROS IR image fragments of a normal-temperature scene around Mexico City from 5 May 2017, namely: (in the upper row) the image fragments from the BIROS TIR band; and (in the lower row) the image fragments from the BIROS MIR band. In both cases, the raw image fragments are shown on the left, the image fragments after the linear radiometric correction are shown in the middle, and the image fragments after the (residual) strip correction including a reconstruction of the “dead columns” are shown on the right.

The striping structures, illustrated in Figure 7, are caused, among other things, by strongly deviating properties and instabilities of certain pixels or even pixel clusters (noise, sensitivity, periodic and spontaneously occurring disturbing signals), which are caused by detector technology. Thus, in addition to the first radiometric processing step, namely the conversion of the digital raw image data into radiance values, the algorithmic recognition and reduction of these interfering structures constitute an important second step in the radiometric calibration process of the FireBIRD IR data.

Compared with the TET-1 radiometric data correction, the radiometric processor of BIROS uses a further development of the so called “striping correction”. This improved correction is based on a variant of the principle of Entropy Maximization (MEM) and can reconstruct defects in images based on the knowledge of the so-called Point Spread Function (PSF) of the MIR and TIR sensor heads at least partially from the surroundings of the disturbance. This takes place without leaving larger artifacts at, e.g., edges with strong contrasts, as was partially still the case with the radiometric processor used in the TET-1 mission. In some BIROS data takes, this even applies to the improved reconstruction of hot spots.

### 3.2. VIS-NIR Push-Broom Camera of the BIROS Main Payload

Satellite sensors for the monitoring and evaluation of HTEs must have, in addition to the MIR and TIR bands, at least two spectral bands in the reflective wavelength range, namely:

- a band in the visual range (VIS), at 0.56–0.72 µm, in order to distinguish fire from solar reflections by pixel-wise ratio formation of the coregistered signals of the VIS band and MIR band, and to eliminate the latter as false alarms; and
- a near-infrared (NIR) band, at 0.79–0.93 µm, to create a cloud mask in daytime images to observe fires in cloud-free areas.

For remote sensing of normal-temperature phenomena (NTP) and for burned area determination, an additional green band at 0.46–056 µm is useful. Therefore, the VIS-NIR camera of the main BIROS payload, incidentally to the VIS-NIR camera of TET-1, is equipped with these three VIS-NIR spectral bands in the reflective wavelength range.

#### Structure and Function of the BIROS VIS-NIR Push-Broom Camera

In the VIS-NIR camera head, as can be seen in the center of Figure 4 and Figure 5 shown in the previous subsection on the bi-spectral IR sensors, three Charge-Coupled Device (CCD)-type linear array detectors are arranged as a hybrid focal unit on the focal plane of the entrance lens of this camera. In front of the Thomson TH 7808 B-type CCD line arrays are mounted their respective spectral filters.

Due to the mounting orientation of the VIS-NIR camera head between the MIR and TIR sensor heads in the multi-spectral camera system of the BIROS payload segment, the longitudinal axes of the three CCD lines are perpendicular to the direction of movement of the satellite track over the Earth during data acquisition in orbit, as illustrated in Figure 2 (also shown in the previous subsection) for a scene scan according to the “push-broom” principle.

The linear ground sampling distance (GSD) of a push-broom camera in the nadir area is determined by the following simple relation:

GSD = p × (h/f)(1)

where:

- p is the distance between two detector elements (pitch);
- f is the focal length of the entrance lens; and
- h is the orbit height of the satellite above the Earth.

The pixel dwell time is calculated according to the formula:

Tdw = GSD/V0(2)

where V0 is the projection of the satellite’s velocity vector onto the Earth’s surface, i.e., the velocity at which the satellite footprint moves over the Earth’s surface. Referring to the GSD of the BIROS VIS-NIR camera of 42 m (see Table 2) and a V0 of 7 × 10^3^ m/s, the pixel dwell time for all three CCD lines of the BIROS VIS-NIR camera is:

Tdw = g/V0 = 42 m/7 × 10^3^ m/s = 6 ms (3)

The VIS-NIR CCD lines are read out at a rate of 6 ms so that no imaging gaps occur during the push-broom scene sampling. The exposure or integration time tint of the VIS-NIR camera is only half of the Tdw with tint = 2.7 ms to keep the pixel smearing in the flight direction low. The three lines (green, red, and NIR) are arranged parallel to each other on a hybrid focal plane at a distance between two lines of d = 10.1 mm. This results in an angle of arc tan d/f = 6.4° for the lines looking slightly forward and backward (with the green and the NIR spectral filter, respectively) to the line—with the red spectral filter—looking in the nadir direction.

### 3.3. Spatial Sampling of the FireBIRD Main Payloads

**Figure 8:**
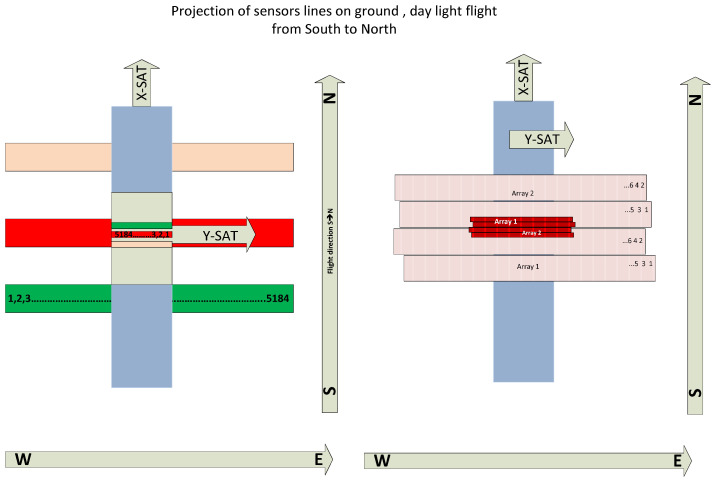
Illustration of the detector line array’s projection onto the ground and the forward movement direction of the TET-1 and BIROS multi-spectral camera systems: left for the VIS-NIR camera with its three CCD lines for the green, red, and near-infrared bands; right for one spectral band of their MIR and TIR bands, each equipped with two (cold-redundant) staggered arrays.

Figure 8 illustrates schematically the projection and forward movement direction of the TET-1 and BIROS main payload detector line arrays onto and over the ground, respectively.

**Figure 9:**
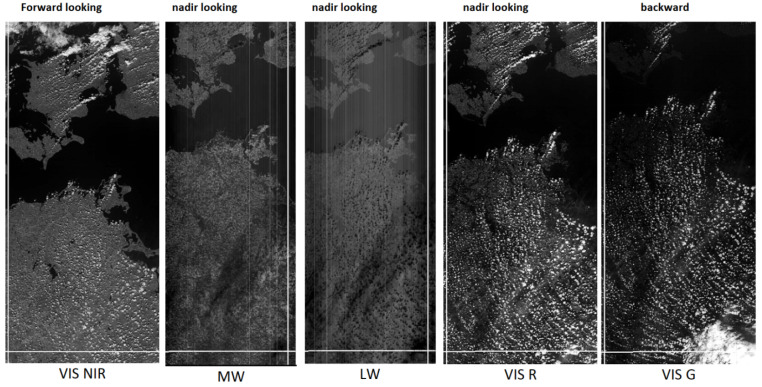
Five image stripes of the TET-1 camera systems, with the Baltic Sea and Ruegen Island in the middle of the forward-looking NIR band on the outer left side. In the center are shown the three nadir-looking bands (from left to right): the mid-wave (MW or MIR) band, the long-wave (LW or TIR) band, and the VIS red band. On the outer right side is shown the backward-looking VIS green band.

Figure 9 shows five TET-1 image stripes with the Baltic Sea and the north of eastern Germany. The shift of the left and right image stripes along the flight direction is due to the 6.4° forward and 6.4 ° backward viewing direction of the NIR and green band detector lines, respectively, as illustrated in Figure 8. The white horizontal “bottom lines” in all five image stripes indicate that the image stripes were registered at the same time, where the time is increasing from the bottom to the top along the image stripe.

## 4. The BIROS Pre-Processing Unit

The FireBIRD concept also includes testing of on-board fire detection and transmission of these compact “fire messages” via a commercially proven orbital communications system to potential users of such information. An “OrbCom Modem”, one of the secondary BIROS payloads, was used for this purpose.

To obtain these “fire messages”, the raw data from the MIR, TIR, NIR, and VIS (red) bands of the multi-spectral camera system have to be” computed” on board of BIROS in a data pre-processing unit (PPU). However, the tasks of the PPU go well beyond this on-board generation of “fire messages”, because it is also used, for all BIROS payloads:

- to receive commands and software uploads transmitted from the FireBIRD ground station for telemetry and command (T/C) to the satellite during its overpass;
- to supervise the payload status and functioning based on the collection of house keeping (HK) data;
- to store all the generated scientific and HK data; and
- to transfer these data to the BIROS transmitters during the satellite’s overpass over the FireBIRD ground station.

## 5. General Design Principles and Features of Small Satellites for High-Resolution Fire Monitoring

By exploring new technologies and methods, initial projects in the university environment showed that small satellites can prepare new experiments and technologies for prospectively larger missions or provide data for complementary scientific questions. Small satellite missions offer the following specific features: implementation with a small budget, shorter development and manufacturing times, smaller but focused user communities, and implementation of new technologies associated with higher technical risks [10].

With respect to the cost drivers of satellite missions, small satellites appeared to be the most suitable solution to achieve challenging mission objectives under low-budget constraints, in particular due to the:

- possibility of so-called “piggy-back launches” together with other satellites, because usually the cost of a piggy-back launch is much lower than that of a dedicated launch;
- accomodation of compact payloads with a high peak power (up to 100 W) and reasonable mean power consumption (<30 W); and
- technical feasibility of small satellite buses that provide sufficient electrical power and volume for these compact payloads with a high peak power and reasonable mean power consumption.

Particularly with regard to the last two points and the associated technical challenges, innovative solutions had to be found for the BIRD demonstrator mission: (a) for the development of a bi-spectral IR camera with cooling machines as a compact sensor system for use in space; and (b) for the development of a small satellite as a complete system with:

- a single-fault tolerance system design;
- a three-axis stabilization of the satellite’s orientation;
- an electrical power supply of 200 W at peak; and
- a total mass of the satellite bus <100 kg.

**Figure 10:**
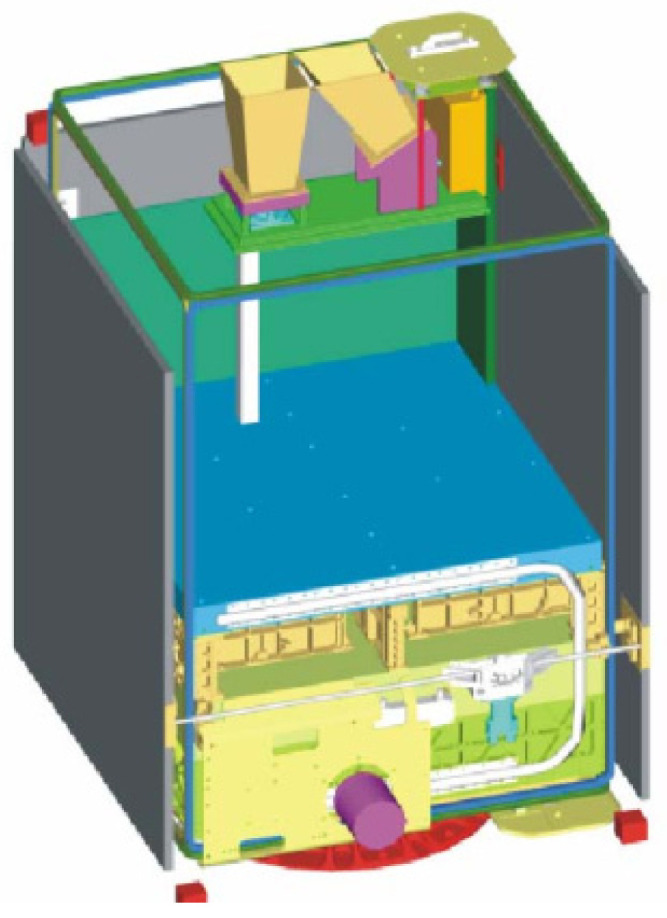
Segment construction of the BIRD satellite (without payload).

**Figure 11:**
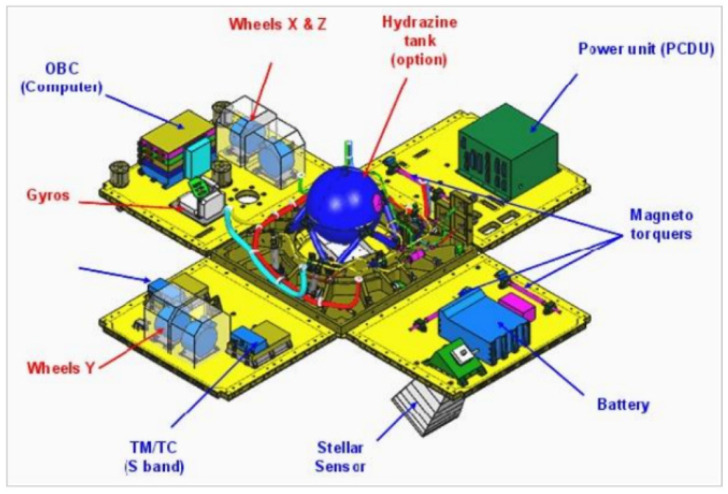
Panel design using the Myriad small satellite as an example [12].

The BIROS satellite bus, like the nearly identical TET-1 satellite bus, is based on the same design principles as the BIRD satellite. The BIRD satellite bus segment structure shown in Figure 10 [11] was new compared with the classic skin-frame structure (or panel structure) as used, for instance, in the French Myriad (see Figure 11) and Portuguese small satellite platforms.

## 6. Overview of the FireBIRD Satellite Busses

The concept of compact segment construction was retained for the TET-1 and the BIROS satellite busses, providing time and cost saving advantages for (i) the DLR space agency ordering the TET-1’s development by German Small and Medium Enterprises /SMEs/ (see https://www.astrofein.com/2728/dwnld/admin/AstroFein_TET_EN.pdf, acessed on 9 December 2021) and (ii) the DLR R&D branch during BIROS’s development at the DLR Institute for Optical Sensor Systems.

**Figure 12:**
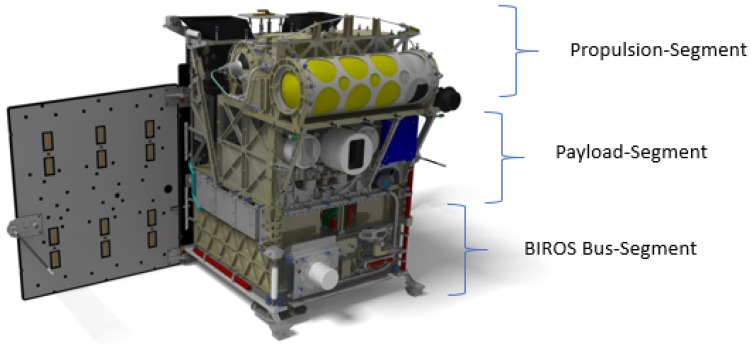
Modular segment design of BIROS comprising bus, payload, and propulsion segments.

This new satellite design approach was comprehensively investigated in a study on “Affordable Space Missions” conducted at DLR Berlin-Adlershof [13] on behalf of the DLR R&D program directorate “Earth Observation”. It was shown that new payloads can be accommodated more easily by a modular segment design combined with a maximum decoupling of the satellite bus and the payload compartment. As a result, there are fewer or no repercussions for or modifications to the satellite bus, which in turn leads to high re-usability of the satellite bus components. Thus, on the TET-1 satellite ten different payloads, including the multi-spectral camera system, could be accommodated in the payload compartment without changes to the bus design. The segment design of the BIRD, TET-1, and BIROS satellites also allows for the highest integration densities, i.e., an optimum utilization of the volume of the overall satellite, which reduced launch costs. The modular segment design of BIROS, which was adopted from BIRD and TET-1, comprising bus, payload, and propulsion segments, is shown in Figure 12.

**Figure 13:**
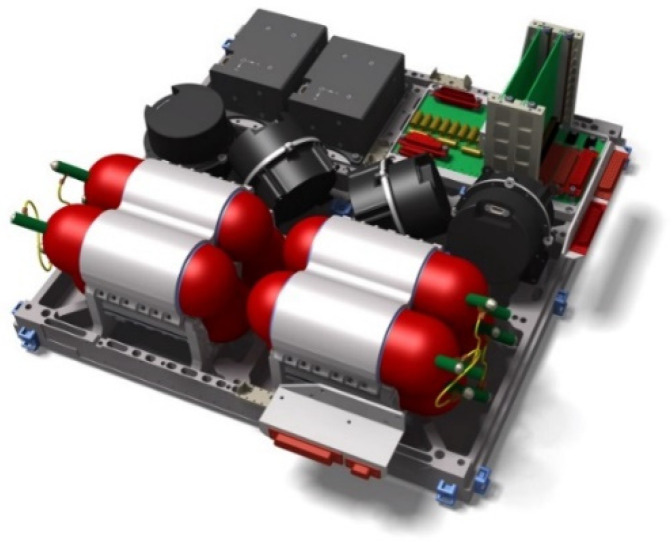
BIROS service segment (DS).

The BIROS bus consists of the Service Segment (in German the “Dienst” segment, DS) and the Electronics Segment (ES). The DS contains the batteries (see Figure 13, marked in red), the four reaction wheels, the Inertial Measurement Unit (IMU), and several electronic boxes.

**Figure 14:**
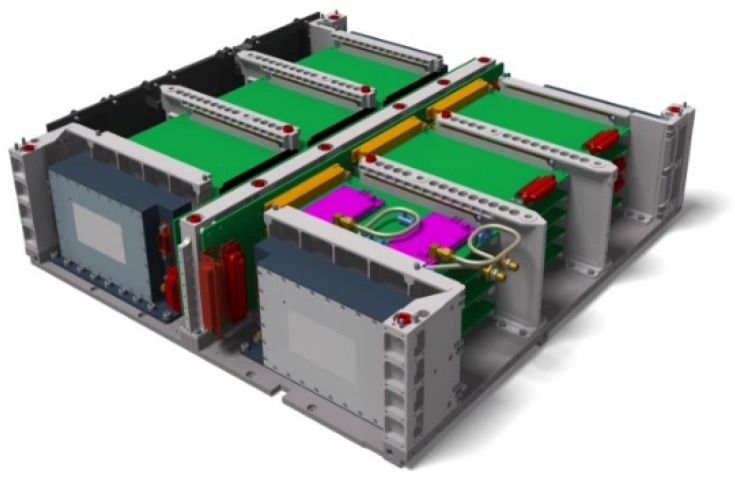
BIROS electronic segment (ES).

The Electronics Segment (ES) has two parts (see Figure 14): the electronics boards for the Satellite Bus Computer (SBC) and the payload computer, i.e., the Pre-Processing Unit (PPU). Figure 13 and Figure 14 illustrate that the components are arranged very close to each other, resulting in (i) a very high degree of integration and (ii) a lightweight satellite support structure that is minimized in terms of the required component dimensions and mass.

**Figure 15:**
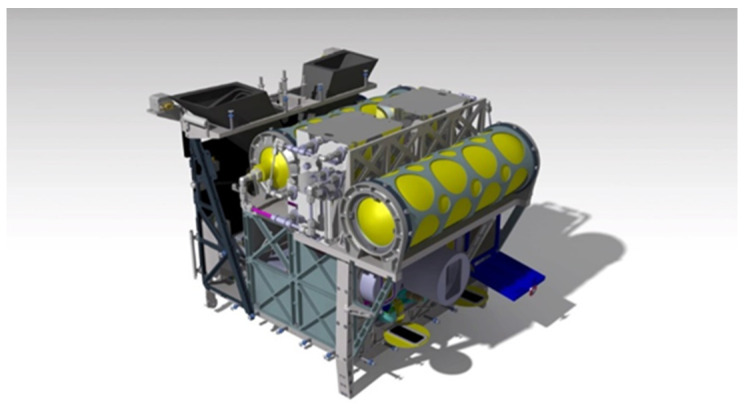
The BIROS propulsion system on top of the payload support structure, the main payload, and a secondary payload: the Single Picosat Launcher (SPL) with its open flap (marked in blue).

The BIROS payload segment (PS) consists of the main payload, i.e., the multi-spectral camera system (see Figure 1, Figure 4 and Figure 5), and several secondary payloads. The BIROS propulsion system is mounted on top of the payload support structure (see Figure 15).

In the following subsections, we will focus on the attitude and orbit control subsystem of BIROS, because this subsystem is very important for the exact pointing of the multi-spectral camera system.

## 7. Attitude and Orbit Control System of the FireBIRD Satellites

### 7.1. Requirements of the Attitude and Orbit Control System of the TET-1 and BIROS Satellites

The main requirements of the attitude and orbit control subsystem (AOCS) for an Earth observation mission in Low Earth Orbit (LEO) are basically derived from the mission analysis and from the pointing requirements of the main payload.

A general mission analysis, as conducted for the FireBIRD satellites, showed that their attitude control (and navigation) must provide a three-axis stabilization of the multi-spectral camera’s orientation within the orbital altitudes of 450 km to 850 km and orbit inclinations from 53° up to ~96° (to secure a sun-synchronous polar orbit).

Thus, the main requirements of the FireBIRD satellite attitude control subsystem are:

(I) The FireBIRD satellites shall be able to adjust the payloads’ viewing directions:
  (a) towards the Earth (nadir, zenith);
  (b) in the flight direction; and/or
  (c) to turn and hold the solar cells towards the sun.
(II) The FireBIRD multi-spectral camera systems shall be able to acquire image data from different viewing directions with respect to the nadir vector. To accomplish this, the following maneuvers shall be feasible:
  - roll around the satellite x-axis (±30°) to increase the Field of Regard (FoR); and
  - pitch around the satellite x-axis (±30°) for stereo imaging.
(III) Design drivers of the attitude control subsystem are the accuracy requirements for the orientation and stabilization of the “Line of Sight (LoS)” of the FireBIRD multi-spectral camera systems, i.e., the main payloads. The main accuracy requirements for attitude control and orientation determination of TET-1 and BIROS are derived from the optical parameters of their main payloads and are detailed in the “Mission Requirement Document” [3] and in the “Technical Specification Bus” [13] and can be briefly described as follows:
  - The orientation accuracy of the satellite: In the so-called Earth-Pointing Mode, the attitude control system must align the satellite with the multi-spectral cameras system, i.e., the +Z axis of the satellite in the body-fixed coordinate system, in the direction of nadir to Earth with an accuracy of 5 arcmin.
  - The jitter of the alignment accuracy for Earth observation shall be less than 2 arcmin/sec.
  - The accuracy of orientation determination relative to Earth: The attitude control and navigation system shall determine the orientation of the satellite relative to the Earth with an accuracy of 24 arcsec.
  - The accuracy of the position determination of an image point of the multi-spectral camera system on the Earth’s surface in the coordinate system WGS 84 (World Geodetic System 1984) shall be <150 m.

### 7.2. The Attitude and Orbit Control Subsystem of the FireBIRD Satellites

This subsection will focus on the attitude control system and will mention the orbit control system only if necessary. The satellite’s motion and orientation directly influence the generation of the remote sensor data as well as the off-line processing of the sensor data to obtain defined data products.

The processing of data from level 0 (raw data) to higher levels requires auxiliary information from the AOCS such as position, orientation, and time. The used AOCS coordinate frames have to be connected to the sensor’s internal frames by a “geometric calibration” allowing for geo-coding, which is usually performed during the off-line data processing on the ground.

During the data acquisition cycles conducted by the FireBIRD multi-spectral camera systems, their Lines of Sight (LoS) must be directed to the Earth’s center, which is secured by the so-called Earth-pointing attitude mode of the AOCS.

**Figure 16:**
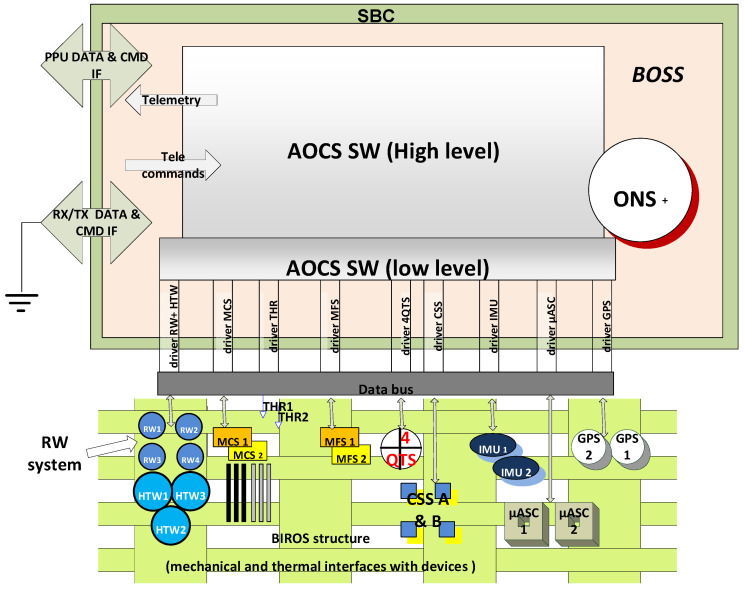
Overview of the BIROS AOCS subsystem.

To perform this attitude mode and further main attitude modes, briefly described in Table 4, the AOCS uses different hardware devices: sensors and actuators. Figure 16 gives an overview of these devices and their connections to the BIROS Satellite Bus Computer (SBC).

The sensors of the BIROS AOCS are:

- two redundant Autonomous Star Cameras (ASCs), i.e., the “Star Sensor Systems” shown in Figure 15;
- two redundant Inertial Measurement Units (IMUs);
- two redundant flux-gate Magnetic Field Sensors (MFSs);
- four redundant Coarse Sun Sensors (CSSs); and
- two redundant Geo Positioning System (GPS) receivers.

The Autonomous Star Cameras (ASCs) and the Inertial Measurement Units (IMUs) provide the precise AOCS orientation data in the body-fixed coordinate system with an accuracy of 24 arcsec and 5 arcsec for TET-1 and BIROS, respectively. The flux-gate Magnetic Field Sensors (MFSs) and the Coarse Sun Sensors (CSSs) provide the coarse AOCS orientation data used, for instance, to align the –Z-axis of the satellite to the Sun to secure the best illumination of the solar arrays.

The actuators of the BIROS AOCS are:

- four Reaction Wheels (RWs); and
- three redundant Magnetic torquer Coils (MCs).

The Reaction Wheels (RWs) and the Magnetic torquer Coils (MCs) allow us to turn and to stabilize the orientation of the satellite in a preselected direction in the body-fixed coordinate system with an accuracy of 30 arcsec.

Comment: 5 arcmin = 1/12 angular degrees is approximately one-sixth of the angular extension of the Sun or Moon seen in the Earth’s sky.

The following Table 5 summarizes the abbreviations give in Figure 16. 

The different clocks used by the AOCS and by the remote sensing image sensor have to be linked in a defined way. The FireBIRD satellites TET-1 and BIROS use some additional status information, such as eclipse state, the availability of AOCS devices during the data take, and the Sun’s direction. In combination with the time-stamped remote sensing data, it is possible to generate a direct geo-coding of the FireBIRD multi-spectral camera data during the off-line data processing on the ground.

## 8. Short Overview of the FireBIRD Ground Segment

**Figure 17:**
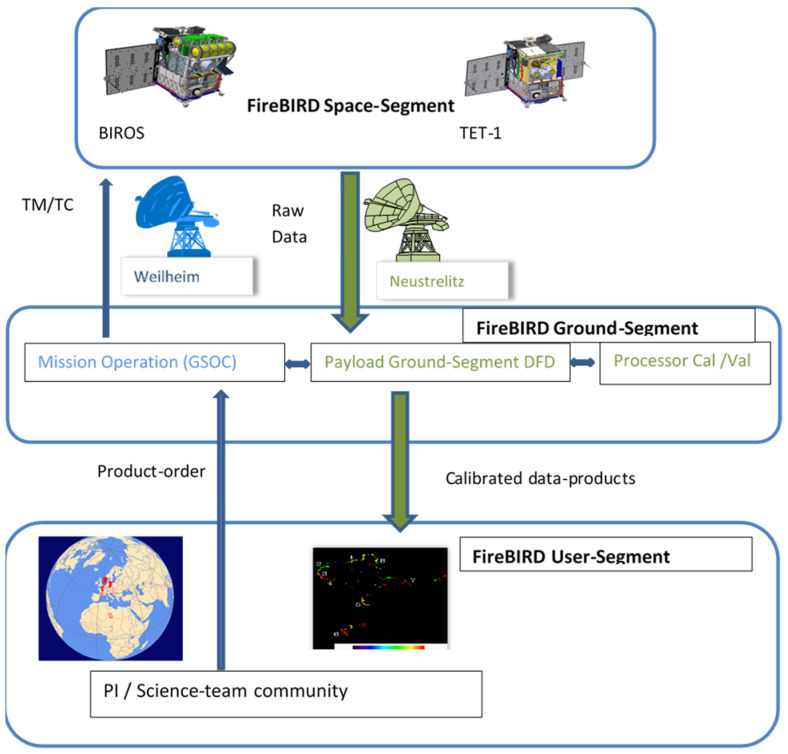
Overview of the FireBIRD ground segment.

The FireBIRD off-line data processing takes place in its ground segment. Figure 17 shows an overview of the FireBIRD ground segment.

The information sequence from the data order, the so-called product order placed primarily by users from the FireBIRD science team, to the FireBIRD data product is shown in Figure 17. The members of the FireBIRD science team can initiate a so-called product order by means of the data order tool, a map-based graphical user interface (GUI) (shown in the lower part of Figure 17). Via the FireBIRD ground control, executed by the German Space Operation Center (GSOC) of the DLR, the corresponding commands are sent to TET-1 or BIROS via the DLR ground stations in Weilheim or Neustrelitz in order to trigger the FireBIRD recordings.

After data acquisition, the raw data from the FireBIRD satellites are sent to the ground via the antennas of the ground stations Neustrelitz, Weilheim, Inuvik, or Gars O’Higgins of the German Remote Sensing Data Center (DFD). At the DFD’s National Ground Segment, the data pockets are sorted according to their specific application and compiled into Level 0 products consisting of image data, calibration data, House Keeping data, and the associated navigation data acquired on board. The data are then processed into radiometrically corrected and geo-coded image data (Level 1 products) and, in a further step of ground-based processing, into thematic products with so-called hot area detection (Level 2 products). Subsequently, the higher-level products (Level 1 and 2) are delivered to the user or to the initiator of the product order via an FTP server, and the Level 0 products are archived together with the parameters originating from Level 1 and 2, such as the location and a summary from the hot area detection, and registered in the EOWEB^®^ GeoPortal (EGP https://eoweb.dlr.de/egp/, accessed on 9 December 2021). Here, users can “log in” and data products can be selected and ordered by using the information on the target location and time of recording.

## 9. Innovative Tools to Increase the Agility of Small Satellites

### 9.1. The BIROS High-Torque Wheels

**Figure 18:**
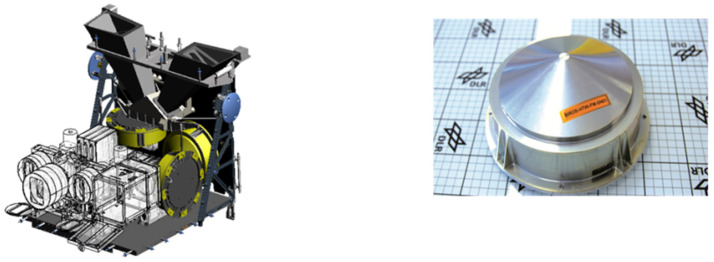
The BIROS High-Torque Wheels (HTWs): one of them is shown on the right side and three of them mounted in the BIROS payload segment are shown on the left side (in the yellow color).

Three High-Torque Wheels (HTWs) [14], mounted as secondary payloads in the BROS payload segment as shown in Figure 18 (and operated as devices of the BIROS AOCS, see the blue marked circles on the left side in Figure 16), were successfully tested on 19 March 2018 over the Lausitz region in East Germany.

**Figure 19:**
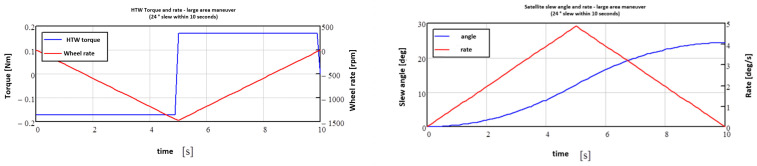
Curves for the torque moment and the slew rate of the HTWs (left diagram) as well as the resulting angular movement and the slew rate of the BIROS satellite around one of its axes (right diagram).

Figure 19 shows the curves for the torque moment and the slew rate of the BIROS HTWs as well as the resulting angular movement and the slew rate of the BIROS satellite around one of its axes.

**Figure 20:**
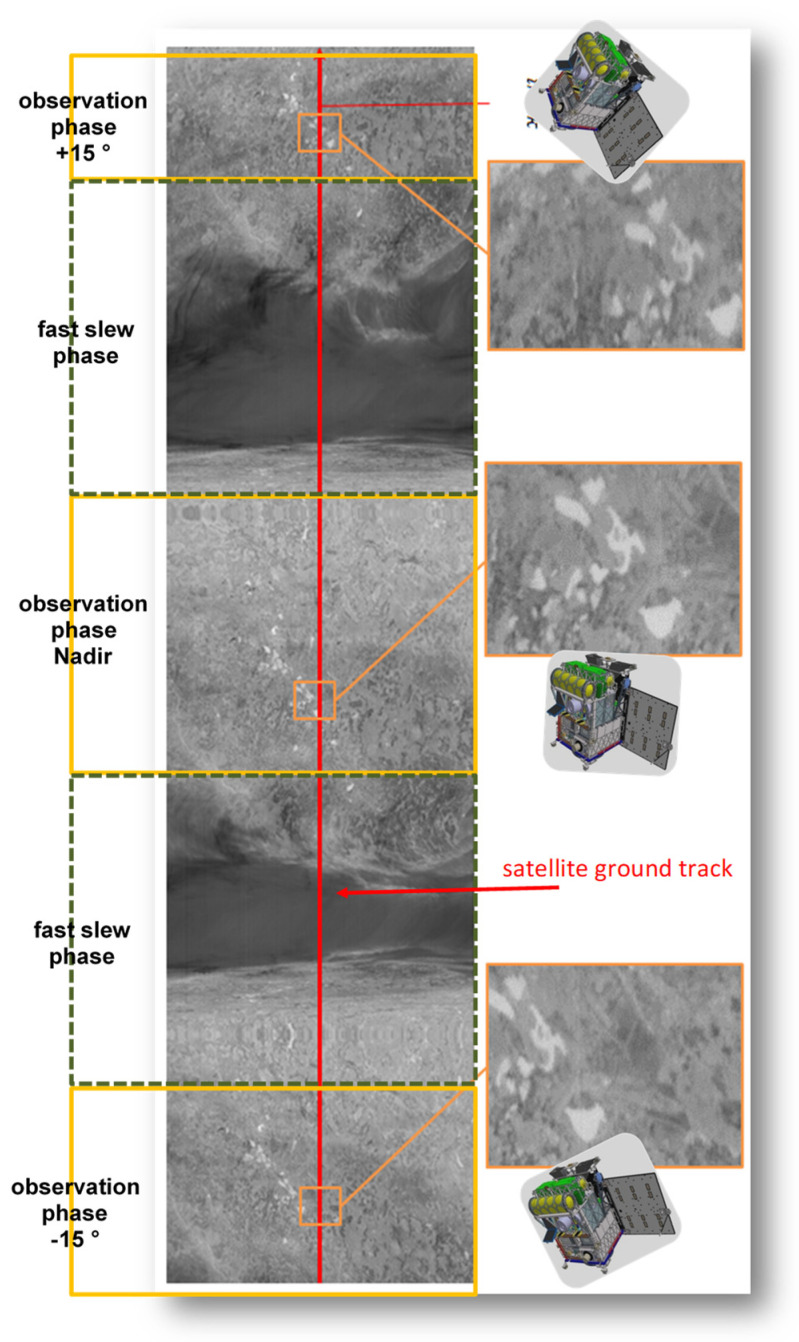
A thermal image sequence acquired on 19 March 2018 at night local time over the Lausitz region in East Germany during attitude maneuvers conducted by the BIROS HTWs and AOCS.

Figure 20 shows a thermal image sequence acquired on 19 March 2018 at 21:15 local time over the Lausitz region in East Germany during several attitude maneuvers of the BIROS HTWs and AOCS. During these maneuvers, the Line of Sight (LoS) of the BIROS main payload performed angular movements or entered into stable, quiet states within the ~8 min duration of all these maneuvers, which were conducted in the following sequence (please see Figure 20 from the bottom to the top):

- looking 15° forward in a quiet state;
- slew 15° from forward to nadir;
- looking nadir in a quiet state;
- slew 15° from nadir to backward; and
- looking 15° backward in a quiet state.

The observed region is a coal mining region, where many former mining pits are artificial lakes now. The right side of Figure 20 shows three magnified thermal image fragments of the scene—without geo-coding—containing the same four major and five smaller artificial lakes whose surface temperatures at 21:15 h local time are higher, i.e., appear brighter, than the mean temperature of the surrounding land surface.

BIROS is the first small satellite capable of recording TIR and MIR data of an Area of Surveillance (AoS) with an ~180 m spatial resolution and three observation angles (nadir and +/−15° along the rack from nadir) within 8 min.

Data of this type can be also used for thermal stereo imaging of clouds, for instance, at night.

### 9.2. The BIROS RGB Matrix Camera

**Figure 21:**
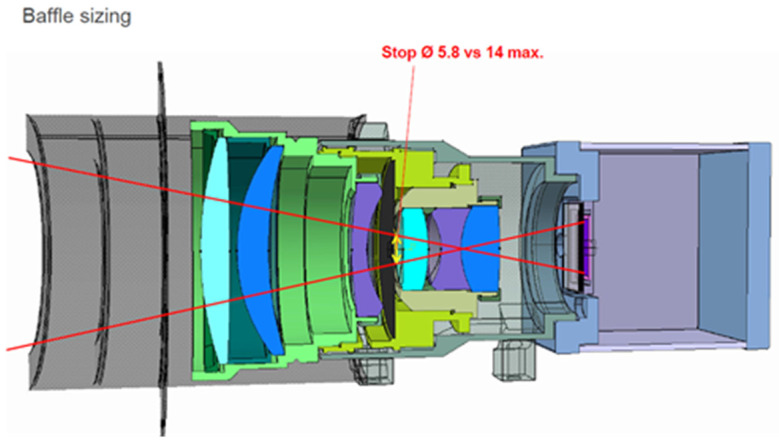
A transparent view of the BIROS RGB camera of type VRm DFC-42 with a baffle in front of the entrance lens.

The Red–Green–Blue (RGB) CMOS matrix camera shown in Figure 21 is a secondary payload of BIROS that was initially used for snap-shooting of a pico satellite launch from BIROS within the experiment named Autonome Visuelle Anflug—Navigation und Target Identifikation (AVANTI) [15]. This RGB camera was also used for Earth imaging when the BIROS satellite was operated in the “Target-pointing” attitude mode, i.e., the camera LoS was fixed to a certain AoS on the Earth’s surface.

Generally, cameras with a matrix detector offer in the push-broom mode interesting advantages compared with a camera equipped with linear array detectors:

- The maximum sampling time Ts-matrix-max may be larger by a factor of N, compared with the Ts-line-max of a linear detector array, where N is the number of detector lines in the matrix array;
- Using, for example, a sampling time of Ts-matrix = Ts-matrix-max/m >> Ts-line-max, m overlapping scene samples can be acquired, digitized, and processed by so-called binning, i.e., an m-fold digital signal accumulation, to improve the signal-to-noise ratio (SNR) by a factor of (m)^1/2^; and
- Stripped spectral filters may be aligned in front of the matrix array segments, which are read out in parallel, to create a multi-spectral camera based on one matrix array detector.

### 9.3. High Agility of Prospective Small Satellites with IR Sensors

**Figure 22:**
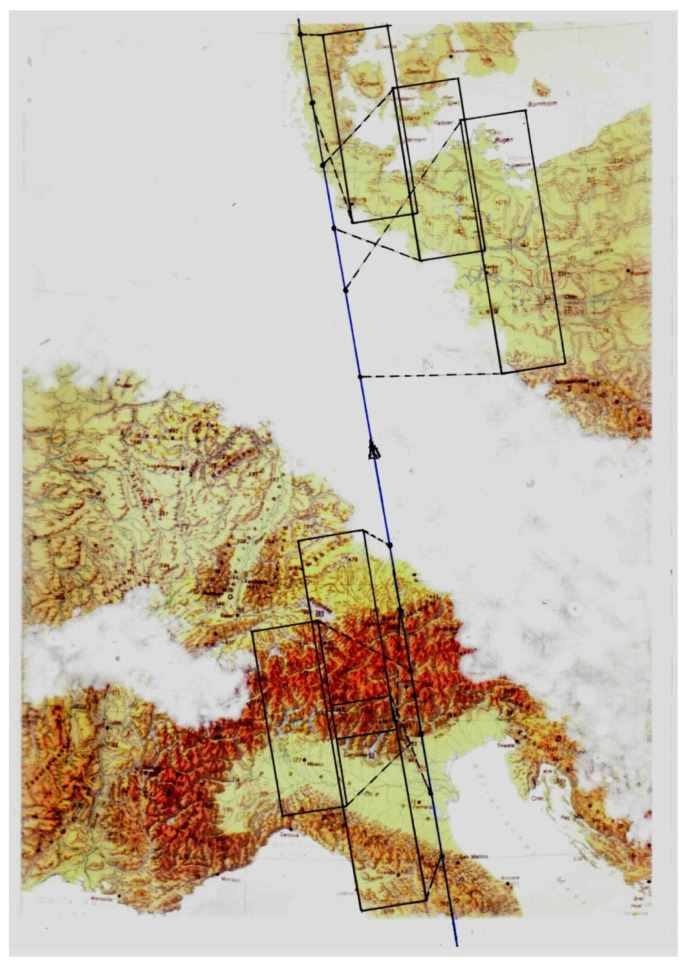
Illustration of intelligent and agile pointing of the Line of Sight (LoS) of a push-broom sensor in a partly cloud-covered scene [17].

Design proposals for compact multi-spectral VIS-NIR and MIR/TIR push-broom sensors based on mega-pixel detector arrays of the COTS type are discussed in [16,17]. The high agility of such a single-satellite sensor system, with (intelligent pointing may be, for instance, the use of information on the cloud cover and cloud-free regions for the pointing of the sensor’s Line of Sight (LoS) in a wide Field of Regard (FoR)) of the sensor’s Line of Sight (LoS) in a wide Field of Regard (FoR), is illustrated in Figure 22. In this example, the LoS of the push-broom sensor is moving forward with a speed of ~27 km/s, which is approximately four times higher than the speed of the satellite over ground (~7 km/s). This high agility is possible due to the use of:

- a cooled CdHgTe matrix detector array in combination with stripped filters in the multi-spectral MIR/TIR sensor (allowing for fast sampling with overlaps also for supplementary data binning); and
- HTWs as the key actuators to enable fast and agile pointing of the sensor’s LoS.

The area covered by the sensor in Figure 22 is approximately two times larger than the area that the sensor would cover in the “classic” push-broom mode, where the sensor’s LoS is moving forward with the speed of the satellite over ground (~7 km/s).

**Figure 23:**
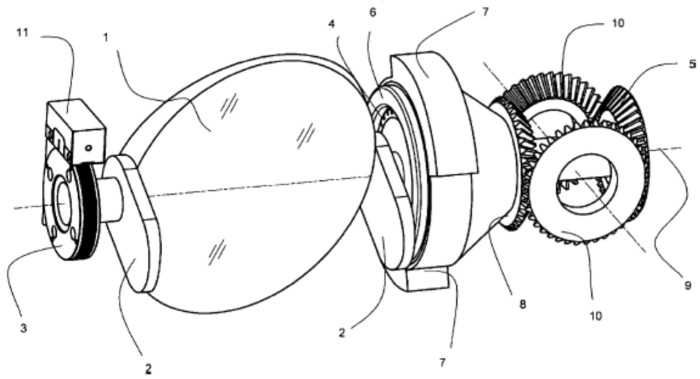
Scan/Tilt Mirror System (STMS) for small satellites equipped with a mechanism for the compensation of the torque moment [18], consisting of the following numbered main parts: 1—mirror, 2—two masses compensating for the mass of the mirror, 3—encoder wheel, 4—rotating part of the drive, 5 and 8—flying cone wheels of the toothed differential transmission, 6—fixed mounted part of the drive, 7—torque-compensating masses, 9—axis, 10—body-fixed cone wheels of the toothed differential transmission, 11—angular encoder.

A Scan/Tilt Mirror System (STMS) for optical payloads on small satellites of, for instance, the BIROS type is shown in Figure 23. This system allows us to conduct either a wide-swath push-whisk-broom scan or to tilt the LoS of a push-broom sensor. The STMS comprises a mechanism for the compensation of the torque moment occurring during the angular movement of the mirror.

**Figure 24:**
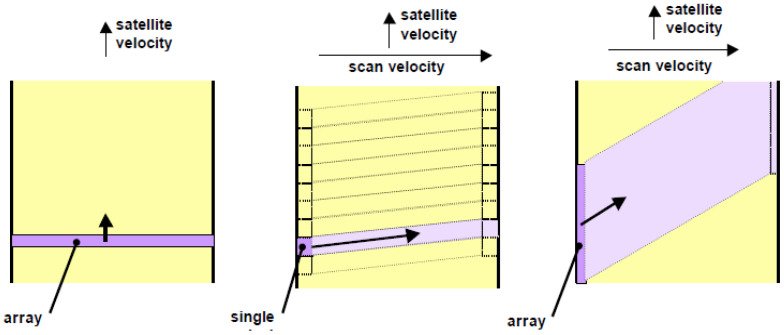
Three typical scan modes of an imaging Earth observation sensor: push-broom (left, with an array detector), whisk-broom (middle, with a single detector), and push-whisk-broom (right, with an array detector).

Figure 24 illustrates the push-broom, whisk-broom, and push-whisk-broom scan modes of an imaging Earth observation sensor.

**Figure 25:**
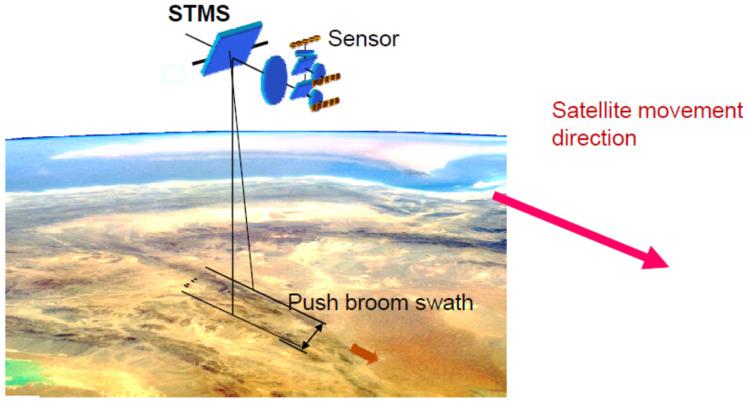
Illustration of the along-track tilt of a push-broom sensor’s LoS by the STMS.

**Figure 26:**
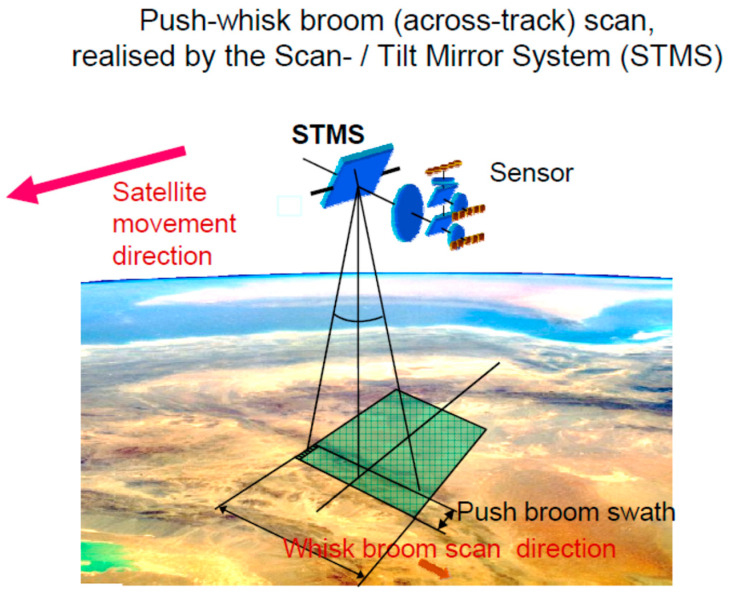
Illustration of the across-track scan of a push-whisk-broom sensor by the STMS.

The utilization of the STMS will allow **us** to implement either an along-track tilt or an across-track wide angle scan, both conducted (i) with one satellite sensor system and—before the start of one of the two sampling modes—(ii) by an initial ~(+/−)90° angular movement of the satellite around its nadir-oriented Z-axis. Figure 25 and Figure 26 illustrate this STMS function for the along-track tilt and the across-track scan, respectively.

## 10. Conclusions and Recommendations

The FireBIRD multi-spectral camera systems scan the Earth in the “push-broom” mode using semiconductor linear array detectors corresponding to the technological state of the art of micro-optoelectronics of the 1990s, when BIRD’s development started. Nevertheless, BIRD and FireBIRD allowed for previously not possible fire dynamic measurements and they provided more detailed, locally based information on fire occurrences than the operational IR sensors currently used for fire applications.

As also concluded in the paper “BIRD and FireBIRD—Pioneering Small Satellite Missions for Infrared Monitoring of Climate Change from Space” prepared for this Special Issue of the *Journal of Imaging* on “Infrared Image Processing for Climate Change Monitoring from Space”, there will be a need for FireBIRD-type IR instruments that operate in a small satellite constellation and provide the following features:

- higher spatial resolution data acquisition without signal saturation from major fires;
- the capacity to augment and validate the more frequent but lower spatial resolution active fire datasets provided by the operational systems, especially for the purpose of detecting smaller and weaker fires, including the derivation of their FRP values; and
- the delivery of active fire data (also with a higher spatial resolution) acquired (i) at different local times and (ii) at local times in the afternoon when fires are at their most severe.

Furthermore, it is recommended that the great progress that took place in micro-optoelectronic technology during the last ~20 years be taken into consideration, especially in the field of the detector development. There are now available so-called commercial-off-the-shelf (COTS) megapixel matrix detector arrays used for High Definition (HD) and Ultra-High-Definition (UHD) military and civil applications, such as:

- Integrated Detector Cooler Assemblies (IDCAs) of Cadmium Mercury Telluride (CMT) photovoltaic matrix arrays for the MIR and TIR wavelength regions with, for example, 1280 × 1024 detector elements; and
- C-MOS and CCD Si semiconductor matrix detector arrays with, for instance, 9000 × 6000 detector elements.

A satellite bus of BIROS-type with a clear segment structure and a payload segment with a volume of approximately 60 × 60 × 60 cm^3^ will allow for the accommodation of (i) a multi-spectral VIS-NIR and MIR/TIR sensor based on mega-pixel matrix arrays, including cryo-cooler(s), together with a Scan/Tilt Mirror System mounted in front of the sensor heads and (ii) three High-Torque Wheels (see Figure 18) as a supplement to the four Reaction Wheels installed in the service segment of the satellite bus.

Table 6 compares and summarizes the TET-1 and BIROS satellites and, for prospective Innovative Infrared Micro-Satellites, some essential design features, such as:

- MIR/TIR sensor arrangements, including the Integrated Detector Cooler Assembly (IDCA),
- on-board data handling and processing; and
- satellite agility.

## Figures and Tables

**Table 1:** Main characteristics of the BIRD sensor system.

	HSRS	WAOSS-B
Spectral bands	MIR: 3.4–4.2 µm (nadir) TIR: 8.5–9.3 µm (nadir)	NIR: 0.84–0.90 µm (nadir and off-nadir) RED: 0.60–0.67 µm (off-nadir)
Focal length	46.39 mm	21.65 mm
Field of view (FOV)	19°	50°
f-number	2.0	2.8
Detector type	CdHgTe arrays	CCD lines
Detector cooling	Stirling, 80–100 K	Passive, 293 K
Detector element size	30 µm × 30 µm	7 µm × 7 µm
Pixel number	2 × 512 staggered	2880
Quantization	14 bit (for each exposure)	11 bit
Ground pixel size	370 m	185 m
Sampling step	185 m	185 m
Swath width	190 km	533 km

**Table 2:** Features and parameters of the multi-spectral camera system of TET-1 and BIROS.

Features and Parameters	Bi-Spectral IR Sensor	VIS-NIR Camera
Wavelength bands (µm)	MIR: 3.4–4.2; TIR: 8–5–9.3	VIS: 0.5 & 0.6; NIR: ~0.8
Field of View (FOV) *	19°	19.6°
Detector type	CdHgTe line arrays, cooled to 71 K for TIR and 80 K for MIR	Charge-Coupled Device (CCD) line array without cooling
Detector element pitch (µm)	30 × 30	7 × 7
Number of detector elements	2 × 512	3 × 5164
Swath width (km)	178	211
Linear pixel size on ground (m)	356	42.4
Sampling step on ground (m)	178	42.4
Pixel dwell time Tdw (ms)	25.4	6.05

* By slewing the satellites up to +/−30° to the right and left of the nadir, this field of view can be directed to selected fire zones or volcanoes within a field of regard of ~700 km.

**Table 3:** Technical specifications of the BIRD MIR and TIR CMT detectors that were used in the IR sensors of TET-1 and BIROS [5].

**Detector**	
Detector operation temperature	80 K
Number of elements	1024
Element Material	CdHgTe, photovoltaic diode
Multiplexer circuitry	CMOS
Element stagger, line to line	15 µm
Array format	512 × 2 elements
Element pitch	30 µm
Spacing between lines	30 µm
Window	Germanium (AR coated)
Field of view	f/2.0
Filter cut-on wavelength	MW: 3.4 µm; LW: 8.5 µm
Filter cut-off wavelength	MW: 4.2 µm; LW: 9.3 µm
Number of outputs	4
Dynamic range	1.6 V
Power consumption	55 mW
Output impedance	55 Ω
Integration capacitors	1.1, 2.2, 2.4, 3.5 pF
**Performance**	
NEΔT (median)	upper limit MW: 45 mK for 4 ms stare time: LW: 40 mK for 1.5 ms stare time.
Number of dead elements	10 maximum
Number of defective elements	50 maximum
Signal uniformity	± 25% of central value
Pixel rate	5.0 MHz max. per output

**Table 4:** Main attitude modes of the FireBIRD satellites.

Attitude Mode	Brief Characterization and Function
Inertial pointing mode	Align the satellite with an inertial frame
Earth pointing mode	Align the satellite with an orbital fixed frame
Target pointing mode	Align a satellite axis with the direction to a fixed target on the ground
Sun pointing mode	Align a satellite axis with the direction to the Sun
Client observation mode	Align a satellite axis with the direction to another spacecraft

**Table 5:** Abbreviations of Figure 16.

BOSS: BIROS Operating System	MCS: Magnetic Coil System
CMD: Command	MFS: Magnetic Field Sensor
CSS: Cors Sun Sensor System	ONS+: on-board Navigation System (+ stands for extended version, comp. with TET-1)
DTA: Data	QTS: Quadrant Sensor
IMU: Inertial Measurement System (Gyro System only)	RW: Reaction Wheel
GPS: Global Positioning System	Rx: Receiver Unit
HTW: High Torque Wheels	THR: Thruster Unit
IF: Interface	Tx: Transmitter Channel
µASC: Star Sensor System	

**Table 6:** Some essential design features of TET-1, BIROS, and a prospective Innovative Infra-Red Micro-Satellite.

Features	TET-1	BIROS	Innovative IR Micro-Satellite
MIR/TIR sensor arragements, including the IDCA	One separate optics array, one linear detector array (an IDCA), and one on-board calibration unit for each IR band		One optics array, one matrix detector array (an IDCA), and one on-board calibration unit for each of the MIR and TIR multi-band sensor heads, or for one MIR/TIR sensor head.
On-board data handling and processing	In both IR bands: supplementary registration of hot pixel signals with a 30 times shorter integration time within the same sampling step, to allow for Hot Area reconstruction during the off-line ground data processing		Registration of multiple overlapping image fragments in all IR bands with at least two different integration times and subsequent band-related digital binning of the data to improve the signal-to-noise ratio (SNR) and to reduce the raw data rate. Fire detection and estimation of the FRP and other fire attributes
Satellite agility	Across-track tilt (up to +/− 30°) of the sensor’s Line of Sight (LoS) must be initiated at least one orbit before the observation	Across- or along-track tilt of the sensor’s Line of Sight (LoS) by 30° in 10 s using three High-Torque Wheels (HTW)	Very fast across- and/or along-track tilt of the sensor’s Line of Sight (LoS) using three HTWs and a Scan/Tilt Mirror System to change the “sampling mode”. This will allow us to tilt the sensor’s LoS to different Areas of Surveillance (AoS), or to conduct wide-swath scanning.

## Data Availability

FireBIRD data is free of charge and publicly available via DLR’s open data archive EOWEB (https://eoweb.dlr.de/egp/, assessed on 9 December 2021).

## References

[1] Briess K., Jahn H., Lorenz E., Oertel D., Skrbek W., Zhukov B. (2003). Fire recognition potential of the Bi-spectral Infrared Detection (BIRD) satellite. Int. J. Remote Sens..

[2] Zhukov B., Lorenz E., Oertel D., Wooster M., Roberts G. (2006). Spaceborne detection and characterization of fires during the bi-spectral infrared detection (BIRD) experimental small satellite mission (2001–2004). Remote Sens. Environ..

[3] Terzibaschian T., Lorenz E., Bärwald W., Halle W. (2013). FireBird Mission Requirement Document.

[4] Oertel D., Zhukov B., Wooster M., Tank V., Lorenz E., Holzer-Popp T., Goldammer J., Martinez S., Siegert F. (2005). ECOFIRE—Study on Scientific Assessment of Space-Borne High Temperature Event Observing Mission Concepts.

[5] GEC Marconi INRA-RED (1996). BIRD IR DETECTOR PROPOSAL Types E3437 AND E3438, Prog. Doc. No. Q3032, Issue 1.

[6] Skrbek W., Lorenz E. HSRS—An Infrared Sensor for Hot Spot Detection. Proceedings of the SPIE’s 43rd Annual Meeting.

[7] Halle W., Fischer C., Terzibaschian T., Zell A., Reulke R., Cree M., Huang F., Yuan J., Yan W. (2020). Infrared-Image Processing for the DLR FireBIRD Mission. Pattern Recognition, ACPR, 2020, Communications in Computer and Information Science.

[8] Dozier J. (1981). A method for satellite identification of surface temperature fields of subpixel resolution. Remote Sens. Environ..

[9] Jahn H., Reulke R. (2000). Staggered Line Arrays in Pushbroom Cameras: Theory and Application. 2000. Int. Arch. Photogramm. Remote Sens..

[10] Sandau R. (2006). International Study on Cost-Effective Earth Observation Missions.

[11] Walter I., Briess K., Baerwald W., Skrbek W., Schrandt F. (2003). The BIRD payload platform, SPIE-Proceedings. Sensors, Systems and Next—Generation SatellitesVI.

[12] Landiech P. (2008). CNES Mini and Macro Satellites Teams. Overview on CNES Micro and Mini Satellites Missions: In Flight, Under Development and Next, Proceedings of the IAA Symposium on Small Satellite Systems and Services (4S), Rhodes, Greece, 26–30 May 2008.

[13] Bärwald W., Schultz C., Eckert S. (2009). OOV TET-1 Technische Spezifikation Satellitenbus TSB.

[14] Raschke C. (2018). Drehmomentengeber zur Hoch Agilen Lageregelung von Optischen Fernerkundungssatelliten. Ph.D. Thesis.

[15] Gaias G., Ardaens J.-S., Schultz C. The AVANTI Experiment: Flight Results. Proceedings of the 10th International ESA Conference on Guidance, Navigation & Control Systems.

[16] Oertel D., Ruecker G., Hartmann M., Hirsch H., Kaiser J.W., Walter I., Wooster M., Zhukov B. Infrared Imaging Sensor Suite—Mission (IRIS-M), 2013. Proceedings of the “1st International Earth Observation Convoy and Constellation Workshop”, ESA/ESTEC.

[17] Ruecker G., Menz G., Heinemann S., Hartmann M., Oertel D. (2015). VISIR-SAT—A Prospective Micro-Satellite Based Multi-Spectral Thermal Mission for Land Applications, 2015, ISPRS—International Archives of the Photogrammetry. Remote Sens. Spat. Inf. Sci..

[18] (2014). Vorrichtung zur Kompensation von Drehmomenten bei Scan-und Tiltspiegelsystemen für Mikrosatelliten, Gebrauchsmusterschrift.

